# Invasive cribriform carcinoma of the breast: A report of nine cases and a review of the literature

**DOI:** 10.3892/ol.2015.2972

**Published:** 2015-02-17

**Authors:** YIZI CONG, GUANGDONG QIAO, HAIDONG ZOU, JUN LIN, XINGMIAO WANG, XIAOHUI LI, YALUN LI, SHIGUANG ZHU

**Affiliations:** Department of Breast Surgery, Yantai Yuhuangding Hospital Affiliated to the Medical College of Qingdao University, Yantai, Shandong 264400, P.R. China

**Keywords:** breast, invasive cribriform carcinoma, ultrasound, mammography, magnetic resonance imaging, prognosis

## Abstract

Nine cases of infiltrating cribriform carcinoma (ICC) of the breast are reported and the clinicopathological features, particularly the imaging findings, are analyzed in the present study. Sonograms revealed that all masses exhibited a hypoechoic internal echo texture (9/9) and that a number of masses presented with an irregular shape (8/9), obscure boundary (5/9), partially microlobulated (5/9) or well-circumscribed (4/9) margins, and an inhomogeneous echo (8/9). Mammographic imaging revealed increased radiological density masses (6/8), and sand-like calcification was not observed in all patients. In two patients, the tumors were mammographically occult. Magnetic resonance imaging performed on one patient revealed a slightly high signal intensity on fat-saturated T1- and T2-weighted images. Following contrast enhancement, a homogeneous early enhancement was revealed with a quick ascent and quick descent time-density curve. Immunohistochemistry revealed that all ICCs expressed estrogen receptor and progesterone receptor, but that none were positive for human epidermal growth factor receptor 2. The Ki-67 labeling index was 3.75% (range, 2–5%) in the tumor tissue. Four patients were treated with mastectomy and the others with breast-conserving surgery. Six clinically node-negative patients underwent sentinel lymph node biopsy; three then received axillary lymph node dissection. Following surgery, three patients received adjuvant chemotherapy, radiotherapy and hormonal therapy, respectively. With a median follow-up time of 38 months (range, 4–70 months), one patient developed local recurrence following breast-conserving surgery; axillary lymph nodes and distant metastases were not observed. This study confirms that this type of carcinoma has unique biological characteristics and a favorable prognosis, but that it remains possible to experience local recurrence.

## Introduction

Infiltrating cribriform carcinoma (ICC) of the breast, which is characterized by a predominant cribriform growth pattern of its invasive component, is a distinct histological type of invasive carcinoma first described by Page *et al* in 1983 (1). The incidence of ICC is reported to range from 0.3 to 3.5% (1–4). The carcinoma has a low frequency of axillary nodal metastases and a favorable prognosis (2). Previous immunohistochemical studies (2,5–7)have revealed that the majority of patients with ICC exhibited estrogen receptor (ER) and progesterone receptor (PR) positive tumors, while human epidermal growth factor receptor 2 (HER2) amplification was rarely observed (5). For this reason, some people recommended that this favorable histotype with luminal tumor may be suitable for no therapy or endocrine therapy alone (8). However, no standard treatment guidelines exist for ICC and thus, treatment is mostly based on that for invasive ductal carcinoma (IDC). Clinically, the tumor usually presents as a mass, but is frequently asymptomatic. The tumor is not usually detected by mammography, but may be identified as a non-malignant mass on sonography. However, few studies have described its radiological features. In the present study, nine cases of ICC of the breast are presented in order to provide additional clinicopathological data; in particular, imaging findings for its diagnosis and treatment.

## Patients and methods

### Patient selection

Nine patients diagnosed with ICC were treated at the Department of Breast Surgery, Yantai Yuhuangding Hospital Affiliated to the Medical College of Qingdao University (Yantai, Shandong, China) between 2007 and 2012. Based on the new definition by the World Health Organization (WHO) (9) and the description by Page *et al* in 1983, all cases were pure ICC. The study protocol was approved by the Human Ethics Committee of the Yantai Yuhuangding Hospital Affiliated to the Medical College of Qingdao University. Informed consent was obtained from all patients prior to the surgery and the examination of the specimens.

### Patient history

The medical records of the patients were retrieved from the hospital registry and the clinicopathological characteristics, including age, menopausal status, family history, laterality, tumor size, lymph node status, hormone receptor status, HER2 status and radiological examinations, were analyzed, as well as the treatment and outcome.

### Pathological examination

The histology of the primary tumor was reviewed by an expert pathologist. The pathological tumor stage (tumor-node-metastasis stage) was assessed according to the criteria established by the sixth edition of the American Joint Committee on Cancer staging manual (10). Specimens were considered to be HER2-positive when they scored +3 by immunohistochemistry or were shown to be positive by fluorescent *in situ* hybridization (9).

## Results

### Patient characteristics

The patient characteristics are shown in Table I. All nine cases were of females. The median age was 55 years (range, 40–79 years) at diagnosis. Four cases were premenopausal and the rest were menopausal. A mass or lump in the breast was the main symptom in all patients. The tumor involved the left breast in four patients and the right breast in five patients. The tumors were mainly located in the upper outer quadrant (5/9). The median size of the tumors was 1.7 cm (range, 1.0–3.2 cm).

### Examination findings

**Figure 1:**
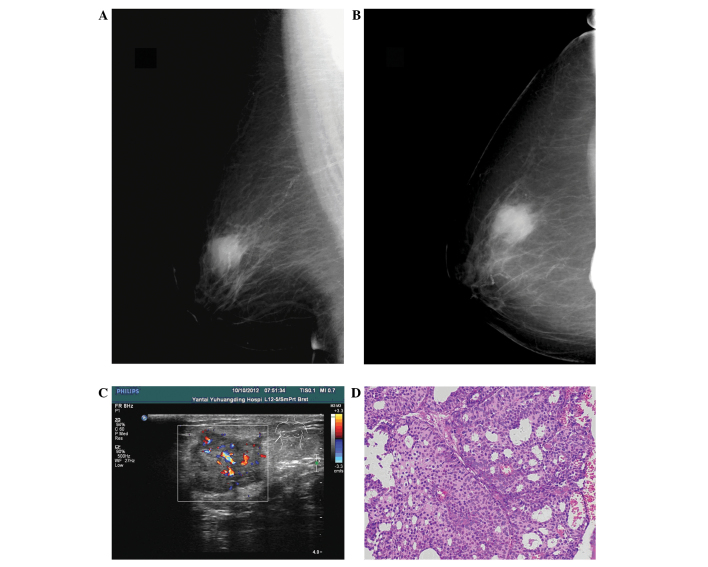
Case 5 from Table I (A) Oblique and (B) axial mammography findings. A high uneven density mass measuring 2.5 cm, with fuzzy boundaries, was observed in the superior external quadrant of the right breast. Enlarged lymph nodes were not observed in the right axilla. (C) Echography imaging revealed a low uneven echo mass range of 2.4×2.0 cm that was lobulated with clear boundaries, crude edges and an irregular shape. Color Doppler flow imaging revealed a rich blood flow signal in and around the mass. (D) Pathological results revealed invasive cribriform carcinoma of the breast (hematoxylin and eosin staining; magnification, ×100).

**Figure 2:**
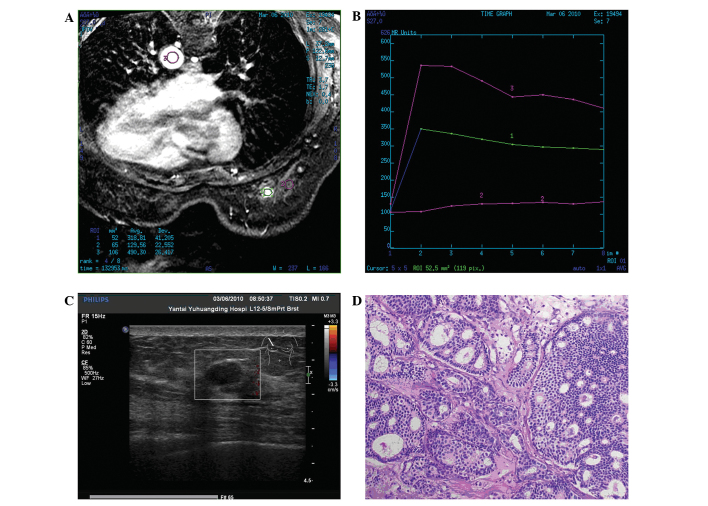
Case 4 from Table I. (A and B) Magnetic resonance imaging findings. The mass measuring 1.3×0.9 cm exhibited a slightly high signal intensity on fat-saturated T1- and T2-weighted images. Following contrast enhancement, (A) a homogeneous early enhancement was revealed with (B) a quick ascent and descent time-density curve. (C) Echography imaging revealed that the low echo mass range of 1.2×0.7 cm was less regular in morphology with a well-defined margin and uneven echo. Color Doppler flow imaging detected no blood flow signal in and around the mass. (D) Pathological results revealed invasive cribriform carcinoma of the breast (hematoxylin and eosin staining; magnification, ×100).

Tumor marker detection revealed that the level of carcinoembryonic antigen in the blood serum was high in one patient (4.01 ng/ml; normal range, 0–3.4 ng/ml) and that the ferritin level was high in two patients (193.0 and 181.7 ng/ml; normal range, 13–150 ng/ml). Pre-operative sonographic findings were available for review for all patients. Sonograms revealed that all masses exhibited a hypoechoic internal echo texture (9/9) and that a number of masses presented with an irregular shape (8/9), obscure boundary (5/9), partially microlobulated (5/9) or well-circumscribed (4/9) margins, and an inhomogeneous echo (8/9). Color Doppler flow imaging revealed markedly increased flow signals in two patients and a resistance index of 0.77 in one patient. Pre-operative mammographic imaging was available for review for eight patients. The tumors in two patients were mammographically occult. The other six tumors that were visible exhibited increased radiological density masses, and sand-like calcification was not observed in all patients (Fig. 1). Magnetic resonance imaging performed in one patient revealed that the mass exhibited a slightly high signal intensity on fat-saturated T1- and T2-weighted images. Following contrast enhancement, a homogeneous early enhancement was revealed with a quick ascent and quick descent time-density curve (Fig. 2).

### Pathological findings

Surgery was the primary treatment for all patients. The frozen section technique was used for intraoperative evaluation, but only 50% (4/8) of patients could be accurately diagnosed. Hematoxylin and eosin (H&E) staining and immunohistochemistry confirmed all cases. Six clinically node-negative patients underwent sentinel lymph node biopsy; three of whom exhibited lymph node metastasis and then received axillary lymph node dissection. Four patients were treated with mastectomy and the others with breast-conserving surgery. The pathological findings revealed five cases with lymph node involvement, one of which exhibited metastasis in more than three lymph nodes. Immunohistochemistry revealed that all ICCs expressed ER and PR, but that none were positive for HER2. An average of 3.75% (range, 2–5%) tumor cells exhibited nuclear staining for Ki-67 (n=4).

### Treatment and follow-up

Following surgery, three patients received adjuvant chemotherapy, three patients received radiotherapy and three patients received adjuvant hormonal therapy. Follow-up was undertaken for eight cases. With a median follow-up time of 38 months (range, 4–70 months), all cases survived without axillary lymph node or distant metastases. However, one patient (case 2; Table I) who received radiotherapy and tamoxifen for six months without chemotherapy developed local recurrence 49 months after breast-conserving surgery. Breast-conserving surgery was performed again for local recurrence at Qilu hospital of Shandong University (Jinan, China). Following surgery, the patient received six cycles of chemotherapy (the specific chemotherapy regimen is unknown) and adjuvant hormonal therapy (letrozole). Subsequent to being followed up for 22 months, the patient was alive with a disease-free status.

## Discussion

ICC of the breast is characterized by a predominant cribriform growth pattern of its invasive component according to the new definition by the WHO (9) and the description by Page *et al* from 1983 (1). Pure ICC is defined as being almost entirely (>90%) of an invasive cribriform pattern, while lesions that demonstrate a predominantly cribriform differentiation with the remaining component limited to a tubular carcinoma (TC) pattern are also included in the category of ICC. Cases with a component that is <50% of a carcinoma type other than TC should be regarded as a mixed type of ICC. In histopathological specimens, this type of carcinoma must be distinguished from other invasive breast carcinomas that exhibit a cribriform pattern, including adenoid cystic carcinoma. Immunocytochemical staining for basement membrane materials or an ultrastructural examination is recommended when accurate diagnosis is difficult (12). In the cases of the present study, it was observed that 50% (4/8) of patients could not be correctly diagnosed by intraoperative frozen section, while H&E staining and immunohistochemistry were able to confirm all cases.

The radiological findings of ICC are not well known, since few studies have described the disease. Stutz *et al* (4) reported that the tumor was mammographically occult in four (50%) cases. The other four tumors all showed as large (20–35-mm) spiculated masses and two contained a few flecks of punctate calcification. Another case report described a circumscribed high-density mass with microcalcifications on mammography, which was described as a borderline lesion on sonography (13). In the present series, all tumors demonstrated an increased radiological density mass with the exception of two (25%), which were mammographically occult, but sand-like calcification was not observed. The ultrasound appearances were not all entirely typical of breast carcinoma. The study by Stutz *et al* revealed that the majority of tumors (3/4) presented as an ill-defined, inhomogeneous solid mass, but without distal acoustic attenuation (4). Another study revealed masses with an oval (2/3) or irregular (1/3) shape, partially microlobulated (2/3) or well-circumscribed (1/3) margins, and a hypoechoic (2/3) or an isoechoic (1/3) internal echo texture. Sonographic assessments were classified as Breast Imaging Reporting and Data System category 4A in two cases and 4C in one case (n=3) (13). In the cases of the present study, all masses exhibited a hypoechoic internal echo texture (9/9) and a number of masses presented with an irregular shape (8/9), obscure boundary (5/9), partially microlobulated (5/9) or well-circumscribed (4/9) margins, and an inhomogeneous echo (8/9). Although the sonographic findings are usually highly suggestive of malignancy, this type of carcinoma may also be shown as a non-malignant mass on sonography. Magnetic resonance imaging is also an essential examination technique for breast cancer. In the present study, the tumor revealed slightly high signal intensity on fat-saturated T1- and T2-weighted images in one patient. Following contrast enhancement, a homogeneous early enhancement was revealed with a quick ascent and quick descent time-density curve. However, homogeneous early enhancement with a delayed wash-out kinetic pattern was also reported (6).

ICC is a well-differentiated neoplasm architecturally, cytologically, ultrastructurally and functionally. The nuclear grade is usually low or moderate, and the ultrastructural features suggest a high degree of differentiation (12). In a previous study, immunohistochemistry revealed that all patients with pure ICC were ER-positive, compared with 20/21 (95.2%) among the mixed ICC cases. PR expression was positive in 26/30 (86.7%) pure ICC cases and 19/21 (90.5%) mixed ICC cases. However, HER2 amplification was rarely observed (5). As in the present cases, all ICCs expressed ER and PR, but none were positive for HER2. Intraductal carcinoma, generally of the cribriform type, and mutifocality are often observed in cases of ICC (1,2,9).

Clinically, the tumor usually presents as a mass, but is frequently occult. For this reason, the lesions are usually larger at presentation, although they grow slowly the majority of the time (3). However, in the present cases, the median size of the tumors (1.7 cm) was not large. The axillary lymph nodes are less frequently involved in ICC than in IDC (1). Venable *et al* (2) reported that the maximal number of metastatic lymph nodes in pure ICC was not more than three, and that there was no marked difference in the positive lymph node rate among the pure ICC, mixed ICC and IDC control groups. As in the present cases, 55.6% (5/9) of patients presented with lymph node metastasis, but only one case involved more than three lymph nodes. Internal mammary node metastasis has also been reported in pure ICC (15).

ICC manifests a better prognosis than that of IDC. The 10-year overall survival rate for ICC is 90–100%, and the outcome of mixed ICC is reported to be less favorable than that of the pure form, but better than that of common ductal carcinoma (1,3,16). In the present study, ICC was identified to be associated with a series of favorable prognostic factors, including a smaller tumor size, less frequent axillary lymph node metastasis, a higher positive rate of ER and PR expression, no HER2 expression and a lower proliferation index. Sand-like calcification, which may be the result of an active secretory process by the tumor cells, was not observed in any of the cases. We believe that these factors lead to its excellent prognosis. Zhang *et al* observed a significantly higher number of positive lymph nodes in mixed ICC than in the pure form. In addition, mixed ICC cases exhibited a higher proliferation index than pure ICC cases, regardless of the positivity rate or the average Ki-67 percentage (5). This result demonstrated that the prognosis of mixed ICC is less favorable than that of the pure form (1,3,15). However, pure ICC could also result in distant (bone) metastasis if untreated (7). In the present study, one patient who received radiotherapy and short-term hormonal therapy without chemotherapy developed local recurrence following breast-conserving surgery, which indicated that adjuvant chemotherapy may play an essential role following surgery.

Surgery was the primary treatment for ICC. Sentinel lymph node biopsy was more suitable due to its lower rate of lymph node metastasis. A molecular classification of breast cancer has been proposed for a better understanding of its biology and treatment. Using immunohistochemistry, the present study found all cases to be of the luminal subtype. The study by Colleoni *et al* (8) recommended that favorable histotypes (e.g., the tubular, cribriform, mucinous and papillary types) with luminal tumors may be suitable for no therapy or endocrine therapy alone. However, such decisions must be made cautiously, as it is still possible to have local recurrence, as in the present case, or distant (bone) metastasis if not treated (7). We therefore hypothesize that chemotherapy and radiotherapy remain necessary for high-risk patients. Endocrine therapy has a significant effect, since the majority of tumors are positive for ER or PR expression.

In summary, the present study described the clinicopathological features, in particular the imaging findings, of nine cases of ICC of the breast. ICC was found to exhibit unique biological characteristics and manifested a good prognosis, as it revealed more favorable prognostic factors. Although local recurrence is possible, when considering the benign course of pure ICC, chemotherapy and radiotherapy may not be indicated in all cases. In the future, larger samples and an increased follow-up time will be required when further examining these findings.

## Figures and Tables

**Table I:** Patient characteristics.

Case no.	Age, years	Menopause	Family history	Laterality	Location (quadrant)	Tumor size, cm	Surgery	Metastatic LNs, n	TNM stage	Immunohistochemistry	Chemotherapy	Radiotherapy	Endocrine therapy	Follow-up, months
1	49	No	Yes	Right	Superior external	1.5	BCS+ALND	5	IIIA	ER(++), PR(+), HER2(+)	CEF^b^	Yes	None	28
2	40	Yes	Yes	Right	Superior external	1.7	BCS+SLNB	0	I	ER(++), PR(++), HER2(−)	None	Yes	Tamoxifen (6 months)	70
3	41	No	No	Right	Superior external	1.1	BCS+SLNB+ALND	1	IIA	ER(++), PR(++), HER2(−)	None	None	None	69
4	69	Yes	No	Right	Superior internal	1.0	BCS+SLNB	0	I	ER(+++), PR(+++), HER2(−), p53(−), Ki-67 3%, CK5/6(−), CD10, p63 focal(+), 34βE12(+)	None	None	None	48
5	68	Yes	No	Right	Superior external	3.2	BCS+ALND	0	IIA	ER(+++), PR(+++), HER2(++)^a^, p53 2%, Ki-67 5%, CK5/6(−), CD56(−), CgA(−), Syn(−)	None	Yes	Letrozole (17 months)	17
6	46	No	Yes	Left	Superior internal	2.5	M+SLNB+ALND	1	IIB	ER(+), PR(+++), HER2(−)	TE^b^	None	Unknown	4
7	79	Yes	No	Left	Superior external	3.0	M+ALND	1	IIB	ER(+), PR(++), HER2(−)	None	None	None	Unknown
8	55	No	Yes	Left	Inferior external	2.0	M+SLNB+ALND	2	IIA	ER(+), PR(++), HER2(−), p53(−), Ki-67 5%	CEF^c^	None	None	53
9	71	Yes	Yes	Left	Inferior internal	1.2	M+SLNB	0	I	ER(+), PR(+), HER2(−), p53 focal(+), Ki-67 2%	None	None	Arimidex (25 months)	25

a Immunohistochemistry revealed HER2(++), fluorescence *in situ* hybridization confirmed HER2(−);

b four cycles;

c six cycles.

LNs, lymph nodes; BCS, breast-conserving surgery; M, mastectomy; SLNB, sentinel lymph node biopsy; ALND, axillary lymph node dissection; ER, estrogen receptor; PR, progesterone receptor; HER2, human epidermal growth factor receptor 2; TNM, tumor-node-metastasis; CEF, cyclophosphamide, epirubicin and fluorouracil; TE, docetaxel and epirubicin.
